# Internal Jugular Venous Pseudoaneurysm in a Patient with Heart Failure and Severe Tricuspid Regurgitation

**DOI:** 10.1155/2017/3592459

**Published:** 2017-05-31

**Authors:** Sujoy Phookan, Patrick T. Strickland, Bishoy Hanna, Gregory R. Hartlage, Ankit Parikh, Stephen D. Clements

**Affiliations:** Department of Medicine, Division of Cardiology, Emory University School of Medicine, 1364 Clifton Road, Atlanta, GA 30322, USA

## Abstract

The differential diagnosis of a lateral neck mass includes a number of possible etiologies. While jugular venous aneurysms and pseudoaneurysms are rare entities, they should be considered in the differential diagnosis of a pulsatile lateral neck mass. We present a case of an idiopathic jugular venous pseudoaneurysm and its association with worsening tricuspid regurgitation in a patient with heart failure with preserved ejection fraction.

## 1. Introduction

The differential diagnosis of a lateral neck mass includes a number of possible etiologies, including vascular disease. We present a case of an individual with a jugular venous pseudoaneurysm that was associated with tricuspid regurgitation. The prominence of the pulsations of the pseudoaneurysm correlated with the severity of the tricuspid regurgitation and the degree of the patient's volume overload.

## 2. Case

The patient was a 73-year-old woman who was admitted for respiratory distress in the setting of heart failure with preserved ejection fraction. Her past medical history was significant for hypertension as well as atrial fibrillation with a permanent pacemaker.

**Figure 1:**
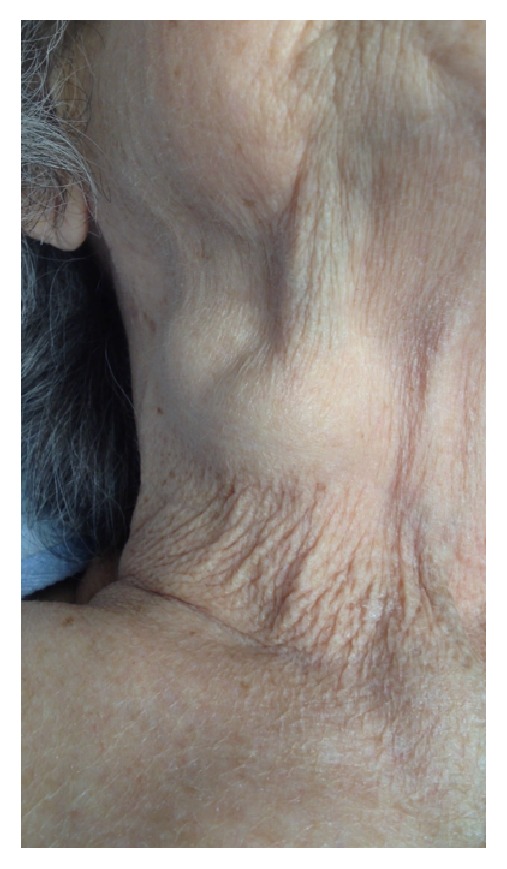
A picture of the neck mass that prompted ultrasound evaluation.

On physical examination, she was found to have a pulsatile right-sided neck mass that was approximately 4 cm in diameter (Figure 1). The mass was soft, compressible, nontender, nonerythematous, and not associated with any overlying skin changes. There was no history of prior right internal jugular venous cannulation or other antecedent neck trauma. The mass was noted to have systolic pulsation with a waveform consistent with large, regurgitant V-waves from tricuspid valve regurgitation. The pulsatility was most prominent in the supine position and decreased as the individual assumed an upright position. Concurrent cardiac auscultation revealed a grade II/VI holosystolic murmur that was loudest over the left lower sternal border.

**Figure 2:**
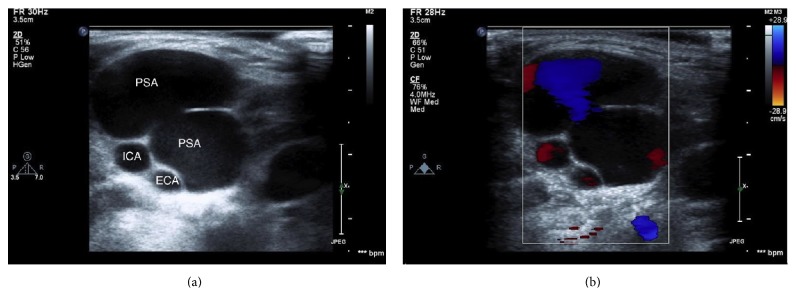
Cross-sectional view of the pseudoaneurysm on vascular imaging. Greyscale is shown in (a) and color Doppler is shown in (b). The color Doppler image demonstrates flow between the lobes of the lesion. In this case, the blue color represents flow towards the transducer. ECA = external carotid artery. ICA = internal carotid artery. PSA = pseudoaneurysm.

**Figure 3:**
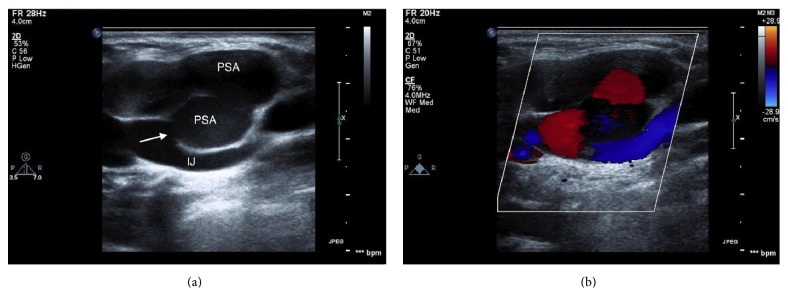
Long-axis view of the pseudoaneurysm on vascular imaging. Greyscale is shown in (a) and color Doppler is shown in (b). The arrow points to the communication between the bilobed pseudoaneurysm and the internal jugular vein. The color Doppler image demonstrates flow into the defect. In this case, the red color represents flow towards the transducer. IJ = internal jugular vein. PSA = pseudoaneurysm.

A transthoracic echocardiogram revealed a normal ejection fraction of 60%, as well as severe tricuspid regurgitation. A vascular ultrasound revealed a bilobed pseudoaneurysm of the internal jugular vein (Figure 2) with an identifiable entrance point and a membrane that separated the two lobes (Figure 3). The pseudoaneurysm became apparent when the patient's tricuspid regurgitation worsened, but the underlying etiology of this defect was unclear.

Diuresis improved the patient's symptoms, the severity of her tricuspid regurgitation, and her central venous pressure. The size of the pulsating neck mass decreased as the patient's hemodynamics improved. At follow-up, several months after hospital discharge, the pseudoaneurysm was no longer visible; of note, the patient was euvolemic at this visit. During a subsequent heart failure hospitalization, a year after the initial one, the pseudoaneurysm reappeared, ostensibly due to worsened right-sided hemodynamics and tricuspid regurgitation. Again, as with the previous hospitalization, diuresis improved the patient's symptoms and decreased the size of the pseudoaneurysm.

## 3. Discussion

The differential diagnosis of a lateral neck mass is broad, including neoplasms, infectious processes, trauma, and vascular and congenital abnormalities [1]. The history and physical examination help to define the etiology of a neck mass and may assist the clinician in choosing the necessary additional imaging modalities. Bedside observation of a pulsatile neck mass should immediately raise suspicion for a vascular anomaly. If careful palpation of the mass reveals a soft, compressible area then a venous abnormality should be considered. Once the mass is identified as vascular in nature, ultrasound can be used to confirm the diagnosis.

Both true venous aneurysms and venous pseudoaneurysms are very rare clinical entities; Calligaro et al. reported no more than 5 in the span of 20 years at their institution [2]. Although they are rare, these conditions may result from prior neck trauma or central venous lines [3]. Interestingly, in this individual, there was no prior history of neck trauma or central venous catheterization. Congenital weakness and vessel wall inflammation have been proposed as possible mechanisms for atraumatic venous aneurysm formation [4, 5]. Tricuspid regurgitation and high central venous pressure, possibly coupled with venous valve incompetence, caused this patient's pseudoaneurysm to manifest as it became engorged with blood. Therefore, this defect served as a barometer of the patient's right-sided hemodynamics.

This patient's vascular defect appeared to be a pseudoaneurysm rather than a true aneurysm because the integrity of the underlying vessel was intact with the exception of a narrow communication between the vessel lumen and surrounding soft tissue containing the lesion. In comparison, a true aneurysm involves dilatation of all three layers of the vessel wall without communication with surrounding soft tissue [2, 6]. To our knowledge, this is the first report of an idiopathic internal jugular pseudoaneurysm in the literature.

Complications of jugular venous aneurysms including thromboembolism or rupture are rare [2]. They do not require anticoagulation and surgical intervention is unnecessary unless there is disfiguration related to lesion progression [2, 7]. Venous aneurysms of the lower extremities, however, can present with recurrent thromboembolism despite anticoagulation and therefore should be excised [7, 8].

## Additional Points

*Learning Objective*. An internal jugular venous aneurysm or pseudoaneurysm should be considered in the differential diagnosis of a pulsatile lateral neck mass. The size of the lesion may correlate with right-sided hemodynamics and the severity of underlying tricuspid regurgitation.
